# Rheumatic heart disease prevalence in Namibia: a retrospective review of surveillance registers

**DOI:** 10.1186/s12872-022-02699-2

**Published:** 2022-06-14

**Authors:** Panduleni Penipawa Shimanda, Stefan Söderberg, Scholastika Ndatinda Iipinge, Ebba Mwalundouta Neliwa, Fenny Fiindje Shidhika, Fredrik Norström

**Affiliations:** 1 Department of Epidemiology and Global Health, Umeå University, 901 87 Umeå, Sweden; 2 Clara Barton School of Nursing, Welwitchia Health Training Centre, Pelican Square, Windhoek, P. o. Box 1835, Namibia; 3 Department of Public Health and Clinical Medicine, Umeå University, Umeå, 901 87 Sweden; 4 Cardiac Outpatient Clinic, Intermediate Hospital Oshakati, Oshakati, Namibia; 5 Department of Paediatric and Congenital Cardiology, Windhoek Central Hospital, Windhoek, Namibia

**Keywords:** Rheumatic heart disease, Acute rheumatic fever, RHD, RF, Namibia

## Abstract

**Background:**

Rheumatic heart disease (RHD) is the most commonly acquired heart disease in children and young people in low and middle-income settings. Fragile health systems and scarcity of data persist to limit the understanding of the relative burden of this disease. The aims of this study were to estimate the prevalence of RHD and to assess the RHD-related health care systems in Namibia.

**Methods:**

Data was retrieved from outpatient and inpatient registers for all patients diagnosed and treated for RHD between January 2010 to December 2020. We used descriptive statistics to estimate the prevalence of RHD. Key observations and engagement with local cardiac clinicians and patients helped to identify key areas of improvement in the systems.

**Results:**

The outpatient register covered 0.032% of the adult Namibian population and combined with the cumulative incidence from the inpatient register we predict the prevalence of clinically diagnosed RHD to be between 0.05% and 0.10% in Namibia. Young people (< 18 years old) are most affected (72%), and most cases are from the north-eastern regions. Mitral heart valve impairment (58%) was the most common among patients. We identified weaknesses in care systems i.e., lack of patient unique identifiers, missing data, and clinic-based prevention activities.

**Conclusion:**

The prevalence of RHD is expected to be lower than previously reported. It will be valuable to investigate latent RHD and patient follow-ups for better estimates of the true burden of disease. Surveillance systems needs improvements to enhance data quality. Plans for expansions of the clinic-based interventions must adopt the “Awareness Surveillance Advocacy Prevention” framework supported by relevant resolutions by the WHO.

## Background

Rheumatic heart disease (RHD) continues to cause premature death and poor quality of life among young populations, with a greater burden on children and women of reproductive age [1, 2]. In 2019, RHD affected about 40.5 million people globally and caused over 300,000 deaths annually, mostly in low and middle-income countries (LMICs) [1, 3].

RHD is an inflammatory heart valve condition, a chronic sequel of Acute Rheumatic Fever (ARF), which is a multisystem disease resulting from an autoimmune reaction presumed to ‘antigenic mimicry’ to certain Group A Streptococcus (GAS) antigenic proteins. ARF develops in about three percent of untreated GAS pharyngitis cases [4–6]. Inflammation in heart valves cause progressive damage with fibrotic changes due to avascularised valvular tissues, leading to chronic RHD disease. The Mitral valve is most commonly affected, as compared to Aortic valve, while mixed valvular involvement is also common [7, 8]. The left sided valves are mostly affected because of the high haemodynamic shear forces, relative to the right sided valves (Tricuspid and Pulmonary). The Tricuspid valve is less structurally involved, however more commonly functionally involved as a haemodynamic consequence of the left heart disease. Presentation ranges from ‘forme fruste’ to severe clinical disease with heart failure, atrial fibrillation, subacute bacterial endocarditis, stroke/cerebrovascular accident, poor maternal outcomes, progressive morbidity/disability with reduced quality of life, and premature mortality [9].

It is a socio-economic disease, and social determinants of health e.g., overcrowding, poor sanitation, and inequitable access to healthcare in poor and socially disadvantaged settings partially attributable to the aetiology of ARF and RHD, in addition to the genetic predisposition [10, 11].

RHD is preventable, by multi-modal interventions i.e., reducing risk factors through improved living conditions, equitable access to health care, and primary prophylaxis. Subsequently, timely diagnosis and secondary prophylaxis of GAS pharyngitis with Benzathine penicillin are crucial to preventing ARF and its sequalae RHD. Cardiac surgery to repair and replace damaged valves, together with lifelong chronic medication, is used to manage the symptoms and prevent severe complications. Benzathine penicillin intramuscular injection has been proven to have superior preventative serum levels compared to oral Penicillin VK, besides improved compliance [12]. Access to the former is however a problem in LMICs.

Limited cardiac expertise, weak surveillance systems, and lack of diagnostic equipment remain a challenge for the RHD prevention and control interventions in LMICs. This contributes to people living with silent RHD for long times until it manifests as severe disease. Another challenge is the lack of true burden of disease estimates on local, national, and international levels to guide evidence-based interventions for the prevention and control of RHD.

### Rheumatic heart disease in the context of Namibia’s health care system

The prevalence of RHD in Namibia has been estimated by a Global Burden of Disease (GBD) study at 1.09% (about 25,200 prevalent cases) [13]. In addition to Congenital Heart Disease (CHD), RHD is ranked among the three most common causes of cardiovascular death in children in the ages 5–14 years in the country [14].

Prevention and surveillance activities, i.e., outpatient register, comprehensive prevention programme, and cardiac surgery, have been established since 2010 along with the Global Rheumatic Heart Disease Registry (REMEDY), a two-year longitudinal study across 14 countries from 2010 to 2012 [15, 16].

Basic RHD care is offered across all healthcare levels, but specialised cardiac care is concentrated at the tertiary national referral centre. Referrals are done with a bottom-up approach from the primary, secondary, and tertiary levels (3-tier system). Cost for public care is heavily subsidised by the government, although out-of-pocket expenditures remains at about 8.2% [17].

Heart surgery is performed for both heart valve replacement using biological and mechanical artificial prostheses, as well as valve preservation strategies (repair). Due to the general paucity of human and infrastructural resources capacity, surgery waiting times can be lengthy, ranging between weeks, months, and even years [14]. Equity-based prioritisation is given to patients grounded on clinical severity and prognosis.

Penicillin remains the primary antibiotic for treating ARF and as secondary prophylaxis for patients with RHD [18]. Lifelong post-surgical treatment includes Aspirin for patients with biological prostheses, and Warfarin for patients with mechanical prostheses. Continuous monitoring of the prothrombin time/international normalised ratio (INR) and appropriate dosage adjustments for patients on Warfarin are done at the cardiac Warfarin clinic [19]. Routinely, progesterone derived transdermal patches have been introduced for women of reproductive age on life-long anti-coagulation therapy. Equally, for these cohort of women, should they opt to start a family, there is tailored package for their needs, in collaboration with the Obstetrics and Gynaecology department.

A gap was identified in data and official reports of the burden of RHD in Namibia, causing uncertainty on the sources of information supporting the GBD report. Therefore, this study aimed to estimate the prevalence of RHD and to assess the RHD-related health care systems in Namibia.

## Methodology

Namibia has two sources of data for people with clinical RHD, a register at the public outpatient cardiac clinic, and an inpatient register from the health information system database for public hospital admission records. The outpatient register contains all patients treated for ARF or RHD at the cardiac department, with data captured upon their first visit to the clinic. In the inpatient register, contains all patients admitted due to ARF or RHD in public hospitals.

We conducted a retrospective descriptive study reviewing data from the two registers from January 2010 to December 2020. The outpatient register recorded 812 patients, of which 20 patients were Angolan citizens, and 74 patients recorded deaths. The inpatient register had 1 463 hospital admissions, which are events rather than the number of patients. Like the outpatient register, it is expected to have a small proportion of foreign citizens, mainly Angolans admitted to public hospitals. Sociodemographic (age, gender, region, hospital of admission) and clinical (valve disease/diagnosis, comorbidities) characteristics were collected and analysed from both sources.

In the outpatient register, heart valve diseases were classified as either (i) Mitral, (ii) Aortic, (iii) Tricuspid, or (iv) a mix. Mitral valve disease was defined as having mitral regurgitation, and or mitral stenosis, or any of these mixed with a tricuspid regurgitation or stenosis. A similar definition was applied for Aortic valve disease. Tricuspid valve disease is defined as having tricuspid regurgitation or stenosis alone. Mixed valve disease applies to a person with both Aortic and Mitral, or the two plus Tricuspid valve disease.

Similarly, valvular diseases in the inpatient register were classified as per the 2016 fifth edition of the International Statistical Classification of Disease and Related Health Problems (ICD-10) manual [20]. Acute Rheumatic Fever was defined as ICD-10 codes I00-I02, codes I05 for Rheumatic Mitral valve disease, codes I06 for Rheumatic Aortic valve disease, codes I07 for Rheumatic Tricuspid valve disease, codes I08 for multiple Rheumatic valve disease, codes I09 for other Rheumatic valve disease, and codes I34-I38 for non-rheumatic valve disease.

Descriptive statistics were performed in STATA 14.2 and Microsoft Excel, which are presented as frequencies and percentages.

Prior to acquiring the data, ethical approval was granted by the Namibian Ministry of Health and Social Services research ethical committee (Reference: 17/3/3 PPS). No individual consent was required from patients to use the secondary data, as there was no direct harm to the patient. The identities of all patients were kept confidential and not revealed in the study.

## Results

**Fig. 1:**
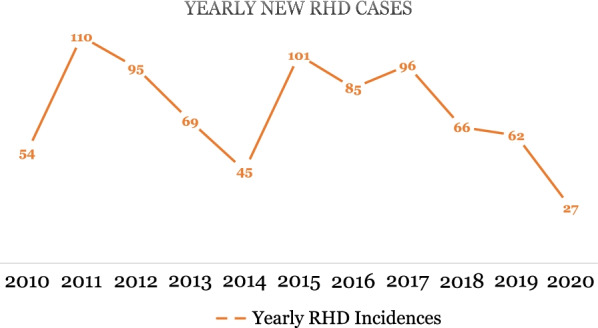
Yearly number of new RHD cases (n = 810). *source*: outpatient register

We found 718 patients regarded as active, which corresponds to a prevalence of 28 cases per 100,000. The highest number of RHD cases (n = 110) were registered in 2011, and the fewest (n = 27) in 2020 (Fig. 1).

Table 1 presents an overview of the distribution of the RHD burden per region from the outpatient register. The highly densely populated north-eastern regions of the country had the highest proportion of the registered cases, accounting for 49% of them. Data from the outpatient register presented in Table 2 show that most patients were young adults less than 30 years old at the time of registration (72%), and a majority were women (65%). Only 467 of the registered patients had data on heart valve impairment recorded, of whom 58% had a mitral valve disease, 19% with mixed valve disease, and 13% with aortic valve disease.

**Table 1:** Number of Rheumatic Heart Disease patients per region

	Population (2016)	RHD cases	RHD deaths	Prevalence/100 000
Namibia	2,550,226	718	74	28
North-Eastern Regions				
Kavango	237,779	32	1	13
Kunene	97,865	31	3	32
Ohangwena	255,510	23	1	9.0
Omusati	249,885	6	1	2.4
Oshana	189,237	235	34	124
Oshikoto	195,165	13	1	6.7
Zambezi	98,849	20	0	20
Central-Western Regions				
Khomas	415,780	213	21	51
Otjozondjupa	154,342	32	2	21
Omaheke	74,629	11	0	15
Erongo	182 402	27	3	15
Southern Regions				
!Karas	85,759	13	0	15
Hardap	87,186	19	5	22

**Table 2:** Characteristics of patients with rheumatic heart disease in the outpatient register (*n* = 812)

	n	%
Sex (n = 808)		
Women Men	523	65
	285	35
Age at Registration (n = 802)		
0–9 years	181	23
10–19 years	250	31
20–29 years	143	18
30–39 years	115	14
40–49 years	36	4.5
≥ 50 years	77	10
Death over 11 years (n = 74)		
Women	35	6.7
Men	39	14
NYHA Classification* (n = 335)		
NYHA I	254	76
NYHA II	45	13
NYHA III	2	6.9
NYHA IV	13	3.9
Valve Affected (n = 467)		
Aortic Valve Disease	59	13
Mitral Valve Disease	269	58
Tricuspid Valve Disease	23	4.9
Mixed Valve Disease	90	19
Unclassified Valve Disease	26	5.6
Treatment & Surgery (n = 791)		
Warfarin	164	21
Penicillin prophylaxis	339	43
Surgery	288	36
Co-morbidities & Complications		
Acute Rheumatic Fever/Rheumatic Fever	19	2.3
Atrial Fibrillation	35	4.3
Congenital Heart Disease	5	0.6
Hypertension	22	2.7
Stroke	14	1.7
Others	15	1.8

^*^New York Heart Association Classification

**Fig. 2:**
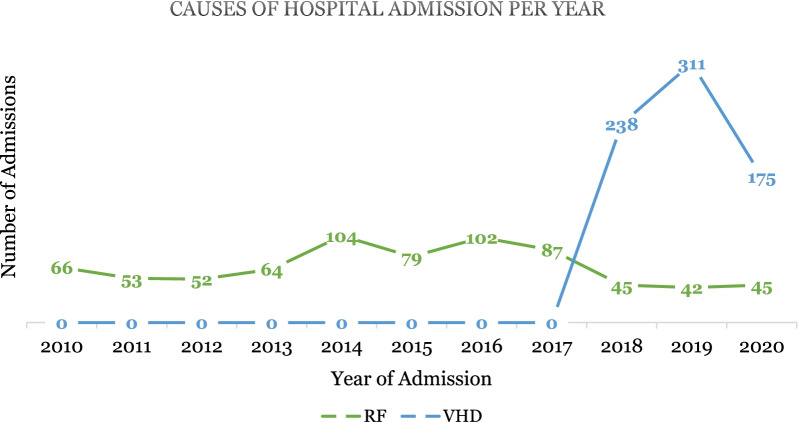
Cause of hospital admission per year (n = 1463). *source*: inpatient register

**Fig. 3:**
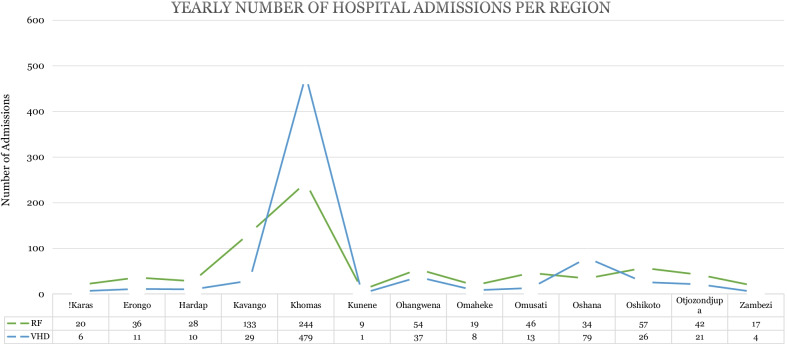
Yearly number of hospital admissions per regions (n = 1463). *source*: inpatient register

Information from the inpatient register is presented in Table 3 and Figs. 2 and 3. Most individuals admitted during this period were young people and, predominantly women. ARF was attributed to 51% of hospital admissions, 24 deaths, and 15 surgeries in this 11-year period. The 724 admissions due to RHD were classified as “other Rheumatic heart disease” (33%), “Rheumatic Mitral valve disease” (5%), “Rheumatic Aortic valve disease” (2%), “Rheumatic Tricuspid valve disease (0.7%), “multiple valve disease (1.1%), and “non-rheumatic valve disease” (8%). The length of hospital admissions ranged between 0 and 335 days (median 4 days). Most of the patients (78%) stayed in hospital between 0 and 9 days.

**Table 3:** Characteristics of patients hospitalised between 2010 and 2021 due to ARF/RF and RHD from the inpatient register (*n* = 1463)

	*n* (%)	RF (*n*= 739)	VHD (*n*= 724)
Sex			
Women	883 (60)	452	431
Men	580 (40)	287	293
Age during Hospitalisation			
0–9 years	266 (18)	149	117
10–19 years	289 (20)	152	137
20–29 years	276 (19)	116	160
30–39 years	219 (15)	97	122
40–49 years	128 (8.8)	60	68
≥ 50 years	285 (19)	165	120
Outcome of Hospital Stay			
Deceased	44 (3.0)	24	20
Discharged	1,358 (93)	677	681
Referred to other hospitals	61 (4.2)	38	23
Diagnoses^a^			
Acute Rheumatic Fever/Rheumatic Fever	739 (51)	–	–
Rheumatic Aortic Valve Disease	31 (2.1)	–	–
Rheumatic Mitral Valve Disease	72 (4.9)	–	–
Rheumatic Tricuspid Valve Disease	10 (0.7)	–	–
Multiple Valve Disease	16 (1.1)	–	–
Other Rheumatic Heart Disease	481 (33)	–	–
Non-rheumatic Valve Disease	114 (7.8)	–	–
Surgery			
None	1 388 (95)	724	664
Surgery	75 (5.0)	15	60
Days spent in Hospital			
0–9 days	1,144 (78)	610	534
10–19 days	219 (15)	87	132
20–29 days	54 (3.7)	23	31
30–above days	46 (3.1)	19	27

^a^Categories defined according to the 2016 fifth edition International Statistical Classification of Disease and Related Health Problems (ICD-10) guideline

Several key areas were identified that could limit the quality of the current RHD surveillance systems.

## Discussion

The study aimed to estimate the prevalence of RHD from the outpatient and inpatient registers between 2010 and 2020 and describe RHD-related healthcare practices in Namibia. Of the total population, we estimated an RHD prevalence of 0.032% from the outpatient register, while the cumulative incidence of RHD was 0.058% in the inpatient register, respectively.

The inpatient register can include two or more posts i.e., hospitalised twice or more during the covered time period, and a few of those have died without it being reported and updated in the outpatient register which usually happens if the patients die in the peripheral regions. According to the routines of RHD care in Namibia, all patients should be seen at the clinic as they are recommended to visit the clinic regularly. We therefore expect there to be only a limited number of diagnosed patients that are not covered in our registers. Thus, based on the two registers, and patients not covered in the register we expect the prevalence to lie within the interval of 0.05% to 0.10%.

An important piece in understanding the burden of RHD and the true prevalence of RHD in Namibia is the undiagnosed cases. The only screening study that has been published from Namibia was among 112 school children where two RHD cases were detected, suggesting an age-related prevalence of 1.8% [21]. This would be higher than the age-related GBD estimate (0.53% for ages 0–14 and 1.51% for ages 15–19) [13]. Echocardiographic screening studies among school children in other sub-Saharan Africa countries reported varying results of silent RHD. For instance, 1.18% among 1 102 school children in Zambia [22], and 0.26% among 4 107 school children in Nigeria [23], had undiagnosed RHD. Systematic reviews have presented a prevalence of latent RHD to be 2.1%, seven to eight times higher than the prevalence of clinically diagnosed RHD [24, 25].

Our interpretation is that the GBD study has included a prediction of the RHD prevalence in Namibia based on both diagnosed and undiagnosed cases. Even if accounting for undiagnosed RHD, we expect, based on results from our two registers and results from screening studies that the prevalence of RHD in Namibia should be lower than the 1.09% presented in GBD [13]. Our guess is that prevalence of RHD in Namibia lies in the interval 0.3–0.9%. We cannot confirm though that the GBD number are representative of people with RHD in Namibia, also considering the possibility of false positive in screening studies. To understand the true burden of disease in Namibia, population-based screening studies are needed. Additionally, understanding the specific local sources of RHD data reported in the GBD study for Namibia will make it easier to understand the validity of the GBD report.

Consistent with the existing literature, we found rheumatic mitral valve disease to be the most common compared to mixed or aortic valve diseases [7, 27, 28]. This is notable for the mitral valve disease related complications i.e., atrial fibrillation and congestive heart failure, that increase the risk of premature deaths, and prolonged years living with disability among the young populations.

Furthermore, a similar finding to the existing literature is that RHD affects mostly young people below the age of 30 years, predominantly women of reproductive age [16, 19]. With the adverse impact of RHD on maternal health, it will be valuable to prioritise investments in interventions for the management of RHD in women [26].

The occurrence of RHD is common in the northern regions dominated by rural areas with limited socioeconomic resources. The findings concur with the reported association between social determinants of health and RHD in LMICs, mainly poor sanitation, overcrowding, and inequitable access to healthcare [11, 29, 30]. Improving living conditions and access to health care in the primordial prevention approach is recommended to reduce regional inequities [14]. Namibia remains one of the most unequal countries in the world with a Gini coefficient of 59.1, and the inequalities are greater in rural areas compared to urban areas [31].

A declining pattern was found in the yearly number of registered RHD cases in the outpatient register, but the cause of the pattern is unclear. A decline in the reporting at the outpatient level could be speculated, as the number of hospital admissions were high in the same time period. The lowest cases were registered in 2020 possibly due to the effect of the Covid-19 pandemic.

### Challenges and future recommendations for RHD-related healthcare systems

Frail surveillance systems persist to challenge the understanding of the true burden of disease in LMICs. In Namibia, we identified key areas that will benefit from systemic and structural changes to strengthen data collection methods. Future recommendations are outlined below to inform planning and changes in Namibia and similar settings.

Assessing the RHD-related healthcare practices, we found that the coordination and integration of the registers is minimal, challenging verification and monitoring. This is a well-known challenge for the surveillance systems in LMICs affecting the data quality and control of RHD [32]. It would have been valuable if we could combine the registers to get a better understanding of the total number of people diagnosed with RHD in Namibia for a more accurate prevalence estimate. The registers lack patient unique identifiers, which would be valuable for combining the registers, and also for better monitoring patients. Introducing patient unique identifiers into the systems and possibly electronic registers is recommended to strengthen them, as well as overall surveillance systems. Findings from a pilot of using RHD electronic registers in Zambia suggested that they are practical and feasible in LMICs [33].

We further recommend continuous professional development training for nurses and doctors at all levels of care and also expanding RHD content in health-related curriculums. In addition, it will be significant to standardise laboratory confirmatory tests for GAS in routine care when treating throat infections. This will improve diagnosis and classification of RHD to improve surveillance of the disease. We suspected possible misreporting in the inpatient register as all the admissions between 2010 and 2017 were classified under ARF, causing 24 deaths and five surgeries. ARF is not likely to be a single indication of surgery. The findings could be related to the limited cardiac expertise in the country and the difficulties in identifying ARF by clinical features [4]. According to the local cardiology experts, the real numbers of RHD patients i.e., surgery cases, could be high than otherwise presented in our study and GBD. Therefore, there is a need to review the clinical records and update the registers for better estimates of the burden of disease.

An expansion of current RHD preventive initiatives to the community level will be significant. Activities are mainly clinic-based with minimal engagement with the community. Financial constraints limit the distribution of printed materials at the clinic, which should be improved to achieve wide awareness. On the other hand, the ministry of health and social services remain committed to improve public RHD-related clinical care with an increasing recruitment of cardiologists and surgeons to support. Our findings add to the renowned challenges experienced in LMICs i.e., policy will towards prevention interventions, delayed implementation, and low financial support [7, 34, 35].

### A.S.A.P. framework for future interventions

We urge the health care system to adopt and apply the universal “Awareness Surveillance Advocacy Prevention (A.S.A.P.)” principles in the planning and implementation of interventions to achieve the resolutions set by WHO and other bodies [36–38].

#### Advocacy

Relevant organisations i.e., high learning institutions, public health organisations, and social groups must collaborate with the clinicians to advocate and mobilise for much-needed investments to expand current interventions to the community-level. Action-oriented research is required to identify key areas and feasible strategies to ensure evidence-based advocacy.

#### Surveillance

This will be valuable to introduce ARF and RHD surveillance forms at primary healthcare facilities as part of the current routine health information systems. Further, it is recommendable to establish collaboration with Information and Technology experts to explore the feasibility of developing and maintaining e-registers at regional levels. Patient unique identifiers must be introduced in the surveillance systems to strengthen data quality and allow for monitoring of patients across different levels of care. This will contribute to a better understanding of the referral systems, follow-up of patients, and overall burden of disease at both patient and healthcare system levels.

#### Awareness

This will be beneficial to appoint focal persons for the awareness component to maintain continuous health education sessions via different media platforms and to keep monitoring and evaluation of all activities related to RHD. It may be cost-effective to incorporate awareness strategies into the existing community health outreach and school health services. Another recommendable option is to involve the community healthcare workers in awareness activities [39]. Namibia has a number of community healthcare workers that are mostly are under and or unutilised.

#### Prevention

The commitment to the treatment and management of RHD e.g., cardiac surgery in Namibia is commendable. However, it will be valuable to prioritise efforts at the primordial and primary prevention levels. Addressing inequities in access to health care will be integral to achieving the prevention targets. Routine care at the primary and secondary healthcare levels must include laboratory testing for group A streptococcal (GAS) bacteria among people with pharyngitis, especially children. Positive cases must receive Benzathine Penicillin intramuscular injection, as it is proven to have superior preventative serum levels compared to oral Penicillin VK [12]. Investigations of the barriers to the supply chains, access, and suboptimal compliance will be crucial to the prevention of avoidable complications for individuals with RHD at a lower cost compared to surgical interventions [30, 40, 41]. Another essential part in the prevention efforts will be continuous education, and task-shifting to use cardiac non-expert practitioners for time detection, treatment, and referral of ARF and RHD. These have been reported to be effective LMICs with limited cardiac expertise [22, 42, 43].

## Limitations

Our study has several important limitations. Our registers should give an accurate understanding of patients within inpatient care and receiving follow-up care. A weakness of our study is that we have not got information from other clinics than the inpatient and outpatient clinic. However, according to the routines in Namibia, all patients should be seen at the clinic and they are recommended to regularly visit the outpatient clinic for follow-up care. We therefore expect at most a limited number of patients to not have visited either of the clinics during our coverage time. We also expect the validity of the registers to be very high, even if there could be patients that have not attended follow-up since 2010. While latent RHD is believed to be high in high-risk populations, our study was limited by the lack of data on latent/silent RHD in Namibia and we therefore are limited to guesses when making estimates of the prevalence of RHD in Namibia. Data validity is an issue in regard to missing data and possible errors for information on patient level for other issues than the diagnosis itself. For instance, in the outpatient register we found 65% of the patients with missing information on the heart valve disease, while the inpatient register recorded a patient admitted for 335 days.

## Conclusion

Using national representative registers with patients diagnosed and hospitalised for RF and RHD over 11 years, we estimate the prevalence of clinically confirmed RHD to be between 0.05% and 0.10% in Namibia, and we expect the prevalence of RHD in Namibia to be lower than previous estimates. Identified shortcomings demonstrates the need to improve and strengthen the surveillance and data collection systems. Recommendations can thus be considered to make the necessary improvements to enhance quality and validity of the registers. Future epidemiological and clinical studies i.e., population-based echocardiogram screening studies, are recommendable to understand the true burden of disease.

## Data Availability

Secondary data used in the analyses was collected retrospectively from health datasets after ethical approval was granted. The datasets are not publicly available, as a separate ethical permission is required to share the data with a third part. The ethical permission is to be obtained from the Namibian Ministry of Health and Social Services research ethical committee by the corresponding author with a reasonable request.

## Abbreviations

ARF: Acute rheumatic fever
A.S.A.P.: Awareness surveillance advocacy prevention
GAS: Group A streptococcus
ICD-10: International statistical classification of disease and related health problems version 10
RF: Rheumatic fever
RHD: Rheumatic heart disease
